# A multi-target brain-computer interface based on code modulated visual evoked potentials

**DOI:** 10.1371/journal.pone.0202478

**Published:** 2018-08-17

**Authors:** Yonghui Liu, Qingguo Wei, Zongwu Lu

**Affiliations:** Dept. of Electronic Engineering, School of Information Engineering, Nanchang University, Nanchang, China; University of Minnesota, UNITED STATES

## Abstract

The number of selectable targets is one of the main factors that affect the performance of a brain-computer interface (BCI). Most existing code modulated visual evoked potential (c-VEP) based BCIs use a single pseudorandom binary sequence and its circularly shifting sequences to modulate different stimulus targets, making the number of selectable targets limited by the length of modulation codes. This paper proposes a novel paradigm for c-VEP BCIs, which divides the stimulus targets into four target groups and each group of targets are modulated by a unique pseudorandom binary code and its circularly shifting codes. Based on the paradigm, a four-group c-VEP BCI with a total of 64 stimulus targets was developed and eight subjects were recruited to participate in the BCI experiment. Based on the experimental data, the characteristics of the c-VEP BCI were explored by the analyses of auto- and cross-correlation, frequency spectrum, signal to noise ratio and correlation coefficient. On the basis, single-trial data with the length of one stimulus cycle were classified and the attended target was recognized. The averaged classification accuracy across subjects was 88.36% and the corresponding information transfer rate was as high as 184.6 bit/min. These results suggested that the c-VEP BCI paradigm is both feasible and effective, and provides a new solution for BCI study to substantially increase the number of available targets.

## Introduction

A brain-computer interface (BCI) is a communication system used for information exchange between the brain and external environment [1]. Without the involvement of peripheral nerves and muscles, such a communication method decodes the intent of users by analyzing and classifying the electrical brain signals, translates it into the control command of an external device and thus builds a direct channel between the brain and the external world. The brain signal can be acquired using both invasive and noninvasive methods. Compared to the former that need to implant electrodes into the brain, the latter do not use intracranial electrodes and thus are much saver. The noninvasive methods include electroencephalogram (EEG), functional magnetic resonance imaging (fMRI) and magnetoencephalogram (MEG), among which EEG records brain signals on scalp and is the most convenient method.

Great progresses in BCI study have been achieved in the past decades [2]. Chen et al. presented an EEG based high-speed BCI speller that achieved an information transfer rate (ITR) up to 5.32 bits/s, the highest ITR at that time reported in BCI studies. This study proposed a novel joint frequency-phase modulation method to tag 40 targets with flicking signals of length 0.5 s and developed a user-specific target recognition algorithm using individual calibration data [3]. Edelman et al. investigated the concept of cognitive flexibility using both a 2-dimensional cursor control task with sensorimotor rhythms and a visual attention task with steady-state visual evoked potential (SSVEP) individually and simultaneously. They found no significant difference in accuracy between the two tasks when performing them alone and jointly [4]. Ortner et al. applied an asynchronous SSVEP BCI for orthosis control. Six untrained subjects showed good control with a positive predictive value (PPV) above 60% and the overall PPV for all subjects up to 78% [5]. Pfurtscheller et al. conducted the study on self-paced operation of a hybrid BCI based orthosis with and without an imagery-based brain switch, which was used to activate a four-step SSVEP–based orthosis only when needed for control and deactivate the SSVEP BCI during resting periods. The combination of the two mental strategies revealed a much lower rate of false positives during resting periods compared to the SSVEP BCI alone [6]. Meng et al. explored the control of a robotic arm to complete grasp tasks using a noninvasive BCI and found that subjects could control the robotic arm with high accuracy for performing tasks requiring multiple degree of freedom [7]. Xu et al. presented a innovative BCI paradigm based on miniature event-related potentials using a space-code division multiple access (SCDMA) scheme to reduce visual fatigue [8]. According to the above impactful papers, The research of BCI technology is switching form theoretical investigation to practical applications.

A BCI can be set up using various paradigms, among which visual evoked potential (VEP) based BCIs have the strength of little user training and high ITR, and thereby receives increasing attention [9]. VEP signals are responses of the brain to visual stimuli and are mainly generated over the occipital lobes. According to the difference in modulation methods of stimulus signals, VEPs can be divided into four categories [9–10], i.e. they are the response to stimulus signals derived from either different time slots (time modulated VEP (t-VEP)), different frequencies (frequency modulated VEP (f-VEP)), different pseudorandom codes (pseudorandom code modulated VEP (c-VEP)), or different locations (space modulated VEP (s-VEP)). Among the four kinds of BCIs, the f-VEP BCI and c-VEP BCI can achieve very high ITRs and thus are the most potential BCIs that will be first applied to clinical practice. Compared to f-VEP BCIs that were intensively developed in the past decades, so far c-VEP BCIs have not yet been thoroughly studied and accordingly more efforts should be made to explore this kind of BCIs.

The first c-VEP BCI was put forward by Sutter in 1984 [11] and was tested 8 years later on an amyotrophic lateral sclerosis (ALS) patient. The result suggested that the subject was able to write 10 to 12 words/min [12]. Since then, the c-VEP BCI had not been paid much attention for a long time. In 2001, Bin et al. built a 32-target c-VEP BCI with the highest ITR among all kinds of BCIs at that time [13]. The average ITR yielded by the BCI was as high as 108 bits/min. Subsequently, several research groups reported important progresses in c-VEP BCI studies. Wei et al. presented a novel grouping modulation based c-VEP paradigm that divides all stimulus targets into several groups and targets per group are modulated with a distinct pseudorandom code and its circularly shifting codes [14]. The results showed that the number of targets and resulting ITR can be increased significantly with sight sacrifice of classification accuracy. The averaged ITR and accuracy rate across subjects yielded by the 48-target c-VEP BCI were 181 bits/m and 91.67% respectively. Wittevrongel et al. introduced a novel decoding algorithm based on spatiotemporal beamforming [15]. They showed that this algorithm significantly outperformed an optimized support vector machine (SVM) classifier for a small number of repetitions of the coding sequence. Riechmann et al. developed a c-VEP BCI for accomplishing everyday tasks such as navigation or action selection [16]. The study demonstrated that this work supports the notion of c-VEP BCIs as a particularly fast and robust approach suitable for real-world use. Waytowich et al. presented a novel c-VEP BCI paradigm that attempted to perform spatial decoupling of the targets and flashing stimuli via spatial separation and boundary positioning [17]. Results showed classification accuracies for non-foveal condition comparable with those for direct-foveal condition for longer observation lengths. Spüler et al. made use of one-class support vector machines for creating templates and error-related potentials for target recognition in order to improve the reliability and classification accuracy of c-VEP BCIs [18].

In existing c-VEP BCI paradigms, all stimulus targets are packaged into one group for stimulus presentation and are modulated by either an original pseudorandom binary code and its circularly shifting codes [11–13,15–18] or different pseudorandom binary codes of the same length [19–20]. For the first method, only one target needs to be trained for creating templates of all targets, whereas for the second one, every target needs to be trained for creating its template. To decrease training time for template creating, usually the first approach is employed for target modulation. However, the available number of targets is limited by the length of modulation codes, which can not be increased arbitrarily because it is also constrained by detection time of targets. For example, if the time lag is 2 bits between two adjacent stimuli, a 63-bit M sequence can be circularly shifted to right for 31 times and provides 32 stimuli at most. This is far from enough for some complex applications such as a word input system. On the other hand, one of the major factors that affect ITR is the number of selectable targets. A high-performance BCI usually has a large number of targets. Thus, how to increase the number of targets is a crucial problem in a c-VEP BCI.

In the previous work, we built a 48-target c-VEP BCI by incorporating the existing first method for target modulation into the second one based on grouping modulation of visual stimuli [14]. It aims to substantially increase the number of stimulus targets and the resulting ITR without significantly increasing the training time. In this study, we extended the number of target groups from three to four, adapted the method for target arrangement from principle of equivalent neighbors to separate placement, and applied four different data preprocessing methods to analyze the c-VEP system. Each target group had 16 stimulus targets and a c-VEP BCI with a total of 64 targets were implemented. Classification results of the experimental data derived from eight subjects verified the the feasibility and effectiveness of the paradigm in increasing the number of stimulus targets and the ITR.

## Methods

### Visual stimulator

**Fig 1:**
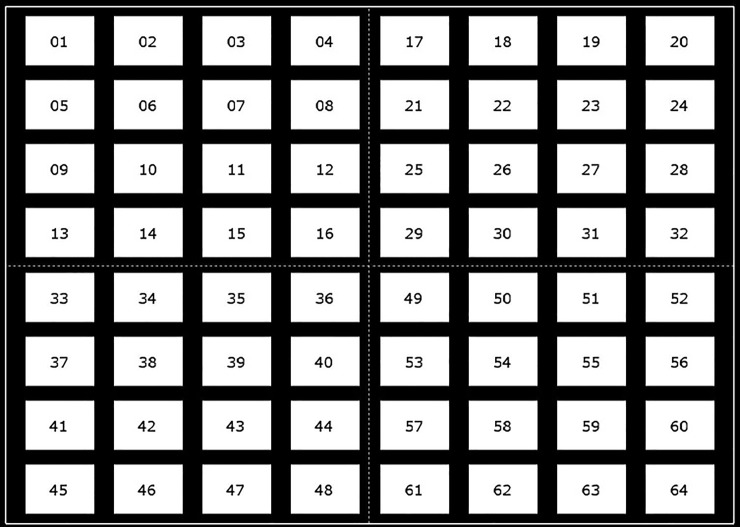
The visual stimulator consisting of four groups of stimulus targets. From left to right and from up to bottom, the number of the four target groups is 1, 2, 3 and 4 respectively. Each group contains 16 stimulus targets arranged in a 4×4 stimulus matrix.

The visual stimulator based on four groups of code modulation is shown in Fig 1. Sixty four stimulus targets are divided into four groups, each of which contains sixteen targets arranged in a 4×4 stimulus matrix. From left to right and from up to bottom, the number of the four target groups is 1, 2, 3 and 4 respectively. The size of each stimulus is a 140×100 pixels. The horizontal and vertical distances of two adjacent stimuli are 40 pixels and 30 pixels respectively. Each group of stimuli are modulated by a distinct pseudorandom binary code (an original code) and its circularly shifting codes. To accurately discriminate among different targets, these modulation codes for all stimulus targets must be orthogonal or near orthogonal to each other [10]. To achieve good separation of targets, any one of the four original codes should have good autocorrelation property so that the sixteen modulation codes in a single group are near orthogonal to each other; Moreover, any two of the four original codes should have good crosscorrelation property so that the modulation codes between different groups are also near orthogonal to each other.

A pair of Golay complementary sequences (G1, G2) [21] and two almost perfect autocorrelation sequences (A1, A2) [22] are selected as the original modulation codes for these four groups of stimuli because they meet the requirements for correlation properties. The four pseudorandom binary codes and their corresponding target groups are listed in Table 1. The length of all the four codes is 64 bits that corresponds to a stimulus period of *P* = 64/60 ≈ 1.066 seconds for the screen refresh rate of 60 Hz. Each original binary code can be circularly shifted for 15 times to the right by the multiple of 4 bits. Thereby, 16 binary codes with different phases are generated and can be used for modulating 16 stimulus targets in a single group. Ones and zeros in these codes denote bright and dark respectively. During stimulus presentation, each target flickers continuously and periodically according its modulation code. When a subject gazes at a target of interest, a c-VEP signal is yielded in the occipital lobe of his brain.

**Table 1:** A pair of Golay complementary sequences (G1, G2) and two almost perfect autocorrelation sequences (A1, A2) used as the four original codes for four groups of stimulus targets.

Group	Modulation code	64-bit pseudorandom binary code
1	G1	1110110111100010111011010001110110111000101101111011100001001000
2	G2	0100011101001000010001111011011100010010000111010001001011100010
3	A1	1000011000100000101011100110100001111001101111110101000110010110
4	A2	0000110001101010000000011011001011110011100101010111111001001101

### Experimental setup

Eight healthy subjects (3 females, aged 21–26 years, mean age: 23 years) with normal or corrected to normal vision participated in the experiments. None of them had a previous history of epilepsy or seizures, which can be induced by flashing stimuli. Two subjects participated in a c-VEP experiment before and were familiar with the experimental setting. Each subject was asked to read and sign an informed consent form before the experiment. The study was approved by the Human Research and Ethics Committee, Nanchang University. After the experiment, subjects were paid for their contribution to the study.

The experimental system consists of a personal computer (Lenovo China) and an EEG amplifier (a Synamps2 system, Neuroscan Inc.) with 64 EEG channels. The computer has a 24-in. liquid crystal display (LCD) monitor and a parallel port linked to the amplifier. The LCD monitor has a refresh rate of 60 Hz and a resolution of1920X1080 pixels. Stimulus presentation was operated in the computer and controlled by the stimulus program developed under Visual C++ 9.0 platform. DirectX (Microsoft Inc.) was employed to ensure the stability of frame-based rendering of stimuli. Event trigger signals yielded by the stimulus program were sent from the parallel port of the computer to the EEG amplifier and recorded on an event channel for synchronizing stimulus presentation and EEG data recordings.

During the experiment, all stimuli were flashed simultaneously and periodically according to their modulation codes. Each subject was seated in a comfortable chair in an unshielded, dimly lit room, approximately 60 cm away from the monitor. They were instructed to focus attention to an intended target, gaze at its center and not to blink as much as possible. Nine Ag/AgCl electrodes over the occipital lobes (P3, Pz, P4, PO7, POz, PO8, O1, Oz, O2) in line with international 10/20 system were used for recording c-VEP signals. The reference electrode was positioned at the vertex. Electrode impedances were kept below 10 kΩ. The EEG signals were digitized at a sampling rate of 1000 Hz.

**Fig 2:**
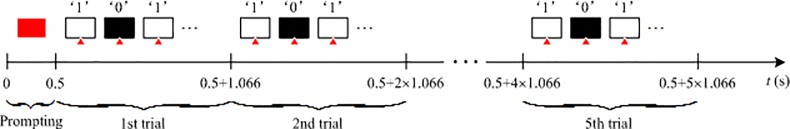
The time structure of testing trials for a target. The first 0.5 s was used for visual cue, during which the target to be tested turned red and the subject should shift his/her gaze to the target as soon as possible. Then the target was continuously tested/attended five trials, during which the red target cue was switched to the red small triangle cue below the target. The time for a trial was equal to one stimulus cycle of the 64-bit modulation codes, i.e. 1.066 seconds for the screen refresh rate of 60 Hz. The data length of a single trial was used for target recognition, i.e. 1066 sampling points for the sampling rate of 1000 Hz. Assume that the first three bits of the modulation code for the tested target is ‘101’. ‘1’ and ‘0’ denote that the target was displayed as ‘bright’ and ‘black’ respectively.

This experiment was divided into two phases: training and testing. The purpose of the training stage was to acquire training data of a reference target per group for constructing a reference template. Any target per group could be selected as the reference target, which was the 11th target in the study. The subject should fixate continuously at each reference target for 100 stimulus cycles (i.e. 100 trials). To avoid visual fatigue, the subject had a 3-minute break between trainings of two reference targets. In testing stage, the subject needed to complete the testing task of all 64 targets, which were tested in a random order. Each test started with a prompting duration of 0.5 s, in which the target turned red and the subject should shift his/her gaze to the target as soon as possible. After the cue duration was over, the red target cue was switched to the red small triangle cue below the target to ensure that the subject was watching the target being tested. The subject was required to fixate continuously on the target for five stimulus cycles (i.e. five trials), and subsequently, the test of next target began immediately. The trial structure for a testing target is displayed in Fig 2.

### Target recognition

#### Canonical correlation analysis

To extract more information related to target recognition, usually multiple channels are used for EEG recordings. Spatial filtering is employed to linearly weight multi-channel signals and integrate them into a one-dimensional signal, so that the signal to noise ratio (SNR) of c-VEP signals can be improved. Canonical correlation analysis (CCA) is an effective method for spatial filtering [23] and is widely used in VEP based BCIs [24–25]. In this study, it is used to create one spatial filter for each target group, which filters the reference template of the corresponding group and single-trial testing data. CCA is a statistical method to study the correlation between two multidimensional variables. The basic principle of CCA is that it creates a pair of linear combinations for two data sets such that the correlation between the two combinations is maximized [26]. Given two multidimensional variables *X* and *Y*, their respective linear combinations, referred to as canonical variants, can be denoted as *x* = *X*^*T*^*W*_*x*_ and *y* = *Y*^*T*^*W*_*y*_. CCA finds the two weight matrices *W*_*x*_ and *W*_*y*_ that maximize the correlation *ρ* between these two canonical variants, which is called canonical correlation. *W*_*x*_ and *W*_*y*_ are determined by solving the following optimization problem

$$\underset{W_{x},\;W_{y}}{\max}\rho (x,y)=\frac{E[x^{T}y]}{\sqrt{E[x^{T}x]E[y^{T}y]}}=\frac{E[W_{x}^{T}XY^{T}W_{y}]}{\sqrt{W_{x}^{T}XX^{T}W_{x}}\sqrt{W_{y}^{T}YY^{T}W_{y}}}$$ (1)

where *ρ* is a one-dimensional vector and *E* denotes the operation of mathematical expectation. The first column of *W*_*x*_, denoted by *w*_*x*_, corresponding to the maximal value in *ρ*, is used as the spatial filter.

#### Spatial filter estimation

In the c-VEP BCI system, the data length used for target recognition is a complete stimulus cycle, which is the data length of single-trials. A spatial filter is estimated with the training data derived from the reference target in each target group. Assume that the reference target *r* in group *i* is continuously attended by *N* stimulus cycles and the c-VEP signal *X*(*t*) is recorded using *C* electrodes. *X*(*t*) is temporally filtered in the frequency band 2–40 Hz because the main energy of c-VEP signal is focused in the frequency band, and then is intercepted into single-trial (i.e. single-cycle) data *X*^*n*^(*t*),*n* = 1,2,⋯,*N*. *X*^*n*^(*t*) ∈ *R*^*C*×*L*^, where *L* is the data length (i.e. the number of sampling points) of single-trials. In this study, *r* = 11, *C =* 9, and *N =* 100. The code length *lc =* 64 bits, the monitor refresh rate *fr =* 60 Hz and the sampling rate of EEG signals *fs =* 1000 Hz, so *L* = *floor*(*lc* / *fr* ⋅ *fs*) = 1066, where *floor* is a Matlab function that denotes rounding towards minus infinite.

Event related potential (ERP) signal $\bar{S}(t)$ is obtained by averaging *X*^*n*^(*t*) across *N* trials

$$\bar{S}(t)=\frac{1}{N}\sum_{n=1}^{N}X^{n}(t)$$ (2)

The ERP component *S*_*r*_(*t*) of the raw c-VEP signals is served as the reference signals of CCA process and can be attained by replicating ${\bar{S}}_{r}(t)$ for N times, $S_{r}(t)=[{\bar{S}}_{r}(t),{\bar{S}}_{r}(t),\cdots ,{\bar{S}}_{r}(t)]$. For the purpose of estimating spatial filters, the single-trial centered data $X_{r}^{n}(t),\;n=1,2,\cdots ,\;N$ are concatenated into continuous data $X_{r}(t)=[X_{r}^{1}(t),X_{r}^{2}(t),\cdots ,X_{r}^{N}(t)]$. *S*_*r*_(*t*),*X*_*r*_(*t*) ∈ *R*^*C*×(*N*⋅*L*)^. Using *S*_*r*_(*t*) and *X*_*r*_(*t*) as the two inputs of CCA, the spatial filter *w*_*xi*_ ∈ *R*^*C*×1^ can be obtained for the *i*th group.

#### Template creating

**Fig 3:**
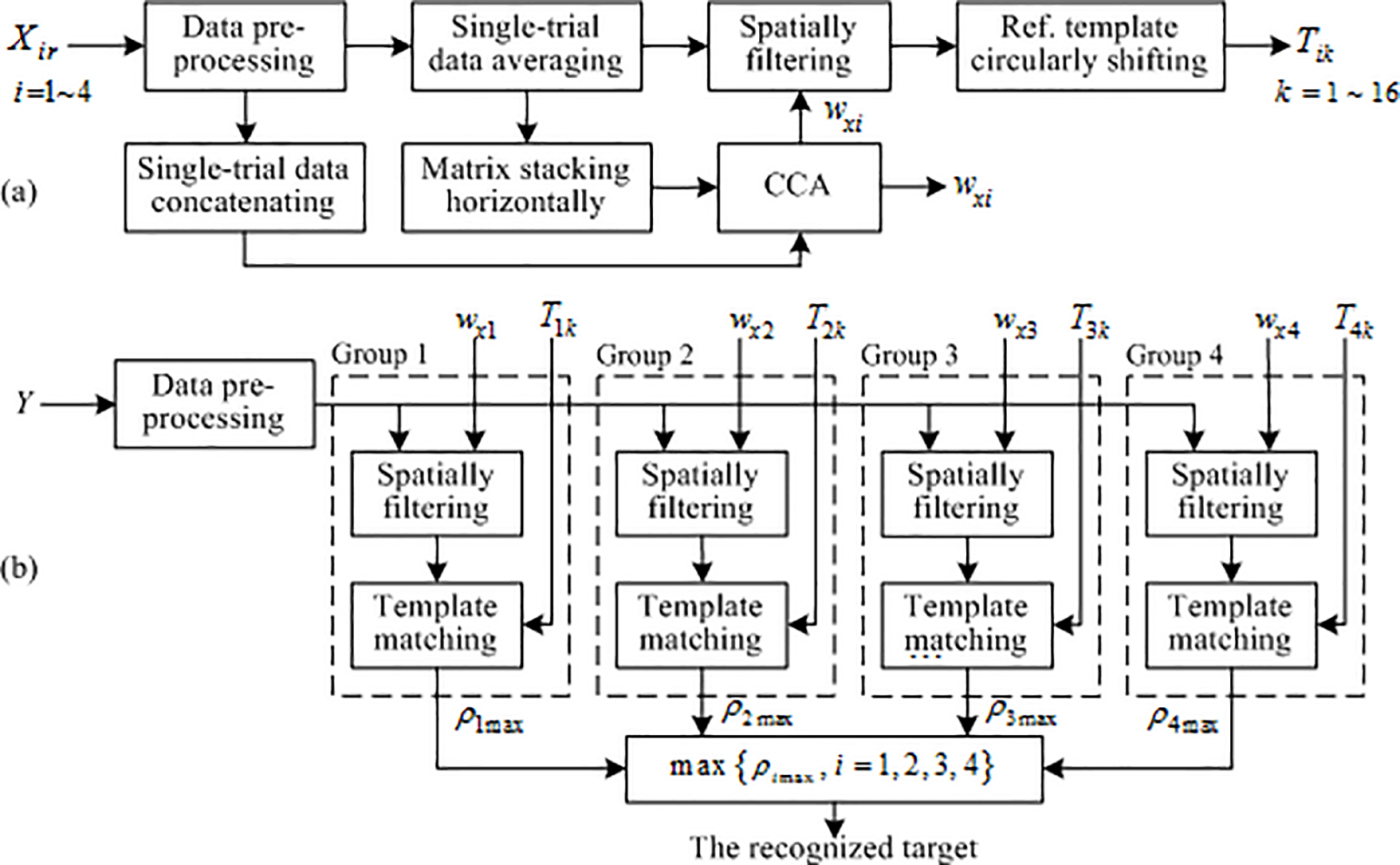
The flowchart of target recognition for the 64-target c-VEP BCI based on grouping modulation of visual stimuli. (a) training stage: Based on training data of the reference target per group, a spatial filter and 16 templates of the 16 targets are calculated for the group; (b) testing stage: Single-trial testing data are recognized by template matching using the four spatial filters and all 64 templates acquired in the training stage.

Template matching is employed for target recognition in the study and is required to create a template for every target in the visual stimulator. The flowchart of template creating in the c-VEP BCI is illustrated in Fig 3(A). The procedure of template creating is as follows:

1. For the *i*th target group, the spatial filter of the group is used to spatially filter the multichannel ERP signal (i.e. the multichannel template) ${\bar{S}}_{ir}(t)$ from the reference target and a one-dimensional reference template *T*_*ir*_(*t*) can be yielded as following
  $$T_{ir}(t)=w_{xi}^{T}{\bar{S}}_{ir}(t)$$ (3)
  where superscript *T* denotes transpose operation.
2. The time lag Δ*τ*_*k*_ between the c-VEP signals of target *k* and the reference target *r* can be calculated according to the number of shifting bits *dt* between their modulation codes
  $$\Delta \tau_{k}=\tau_{k}-\tau_{r}=floor(dt\cdot fs/fr),\;k=1,2,\cdots ,16$$ (4)
3. Templates for all other targets in the group are generated by circularly shifting the reference template according to the following equation
  $$T_{ik}(t)=T_{ir}(t-\Delta \tau_{k}),\;k=1,2,\cdots ,16$$ (5)

#### Template matching

Target recognition is done by template matching between a testing signal and all templates in a single target group and then comparing the best results of all four groups. Template matching uses a similarity measure to match a signal to a template and is widely used in image processing [27–28]. It includes approaches such as normalized cross correlation (NCC), sum of absolute difference (SAD), sum of square difference (SSD), etc. In this study, the correlation method is used for target recognition. The flowchart of target recognition in the c-VEP BCI is illustrated in Fig 3(B).

Assume that a single-trial multichannel testing signal be *Y*(*t*) ∈ *R*^*C*×*L*^. It is temporally filtered in the frequency band 2–40 Hz and then is spatially filtered using the four spatial filters *w*_*xi*_ yielded in the training stage, where *i* = 1,2,3,4 is the number of groups. Four one-dimensional data vectors are generated by spatially filtering, $y_{i}(t)=w_{xi}^{T}Y(t)$. For each target group *i*, 16 correlation coefficients *ρ*_*ik*_ between *y*_*i*_(*t*) and 16 templates *T*_*ik*_ are calculated as follows

$$\rho_{ik}=\frac{\langle y_{i}(t),T_{ik}(t)\rangle }{\sqrt{\langle y_{i}(t),y_{i}(t)\rangle \langle T_{ik}(t),T_{ik}(t)\rangle }},\;k=1,2,\cdots ,16$$ (6)

where 〈*x*,*y*〉 denotes the inner product of two vectors *x* and *y*. The maximum of *ρ*_*ik*_ for the *i*th group is $\rho_{i\max}=\underset{k}{\max}\;\rho_{ik}$. The target with the maximum of the four maximal coefficients is decided as the attended target

$$ST=\underset{i}{\max}\;\rho_{i\max}$$ (7)

### Performance evaluation

#### Information transfer rate (ITR)

Most BCI studies are conducted towards improved ITRs because it is the most important metric for BCI performance. The ITR in bits/minute defined by Wolpaw et al [1] is calculated as follows

$$\mathrm{ITR}=\left(\log_{2}M+P\log_{2}P+(1-P)\log_{2}\left[\frac{1-P}{M-1}\right]\right)*\left(\frac{60}{T}\right)$$ (8)

where *M* is the number of targets, *P* is the classification accuracy of targets, and *T* in seconds/selection is the average time for a selection. Classification accuracy is defined as the ratio of number of trials classified correctly to total number of trials. The time for target selection includes the time for gaze shifting and the time for visual stimulation.

#### Signal to noise ratio (SNR)

Unlike f-VEP analysis, so far there has not been an established approach to define SNR of c-VEP signals. In this study, a similarity index (SMI) [29] was adopted as SNR measure, which is the power ratio between an event-related potential (ERP) signal in a test trial and the residual. Let $\bar{X}$ be the average of all training trials and *x*_*i*_ be the *i*th testing trial. The SMI in decibels (dB) is defined as

$$\mathrm{SMI}=10\;\log_{10}(\sigma^{2}(S)/\sigma^{2}(N))$$ (9)

where $S=\frac{{\bar{X}}^{T}x_{i}}{{\bar{X}}^{T}\bar{X}}X^{T}$ is the orthogonal projection of *x*_*i*_ onto $\bar{X}$, and $N=x_{i}-\bar{X}$ is the residual part. A large SMI implies that the testing trial is similar to the average of training trials $\bar{X}$. Thereby, SMI can be used to approximate the SNR of a single-trial c-VEP signal [30].

### Statistical analysis

Wilcoxon signed-rank test for a statistical analysis of median difference significances was applied to investigate the effect of different number of targets and different data preprocessing methods on system performance, because the performance indices were not normally distributed. The paired test was conducted at 95% confidence level.

## Results

### Auto- and cross-correlation of c-VEP signals

**Fig 4:**
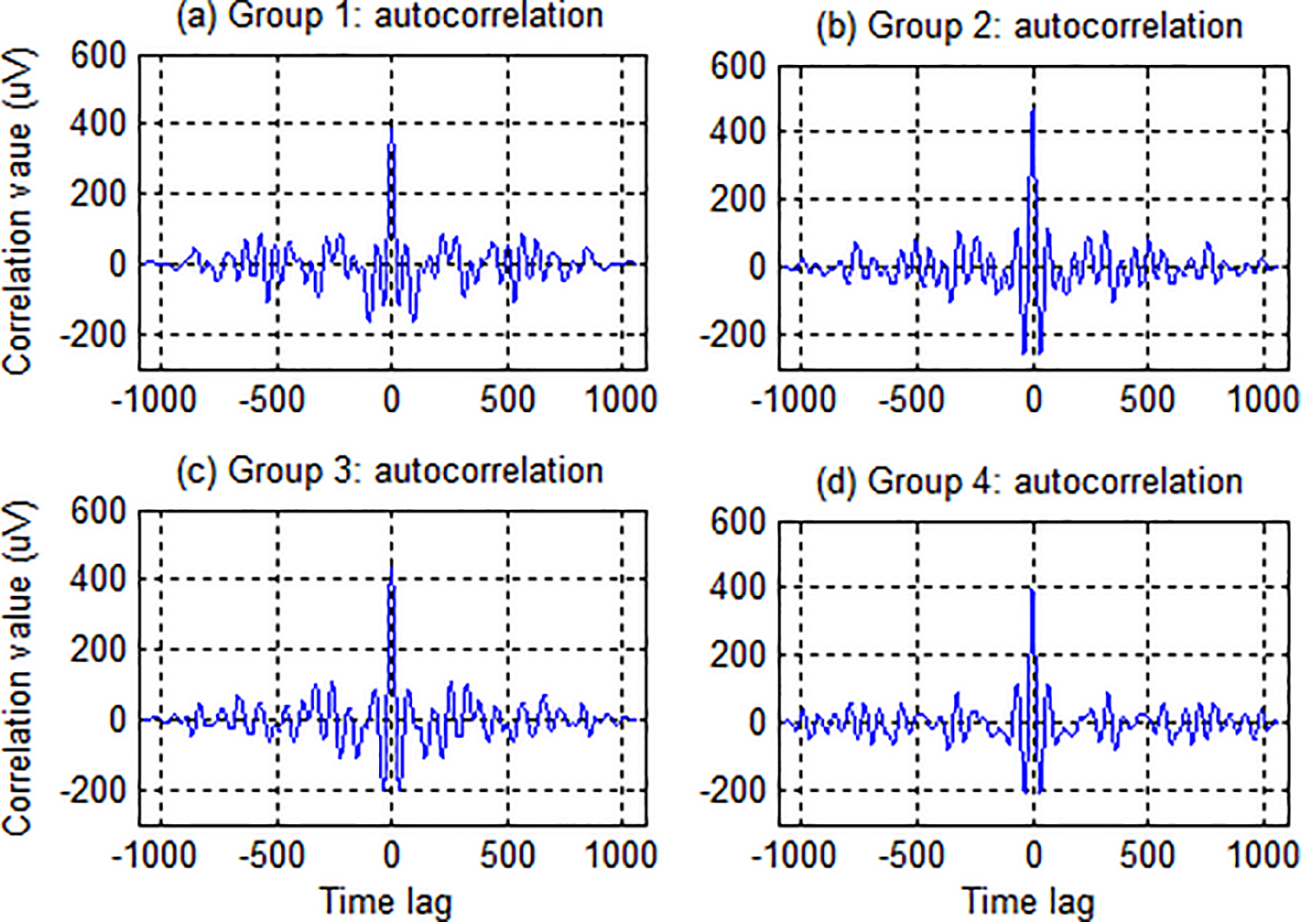
Autocorrelation functions of the four reference templates derived from a representative subject.

Although the visual stimuli in the c-VEP BCI are all uncorrelated as binary signals, this does not guarantee that the responses to these stimuli are also uncorrelated because the brain is a nonlinear dynamic system. Thereby, the separability of stimulus targets depends actually on the orthogonality of stimulus responses (i.e. c-VEP signals) rather than the modulation codes. Fig 4 shows the autocorrelation functions of the four reference templates derived from a representative subject. It is clearly seen from the figure that each of the four autocorrelation functions had a sharp central peak at time lag 0 and low sidelobes elsewhere. The ratio of the central peak to the maximal sidelobe was large enough to ensure accurate discrimination of different targets in a single group.

**Fig 5:**
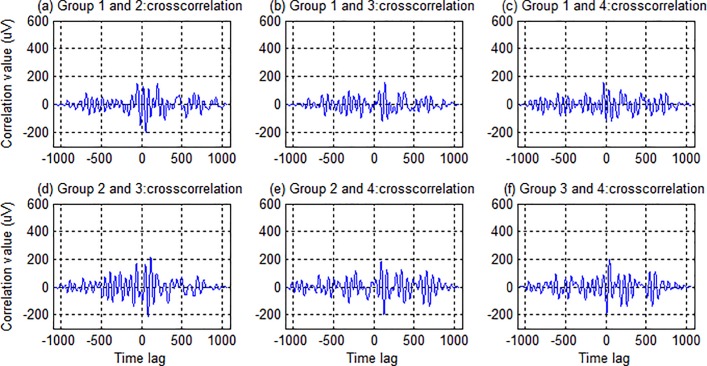
Crosscorrelation functions between any two of the four reference templates derived from a representative subject.

Fig 5 illustrates the crosscorrelation functions between any two of the four reference templates derived from a representative subject. It is observed that the crosscorrelation functions had small values at all time lags compared to the central peaks of autocorrelation functions in Fig 4. This means that targets in different groups is easily discriminated as well. Thereby, the four original modulation codes guarantee in principle the good separability of different stimulus targets among all the four groups.

### Amplitude spectrum and SNR

**Fig 6:**
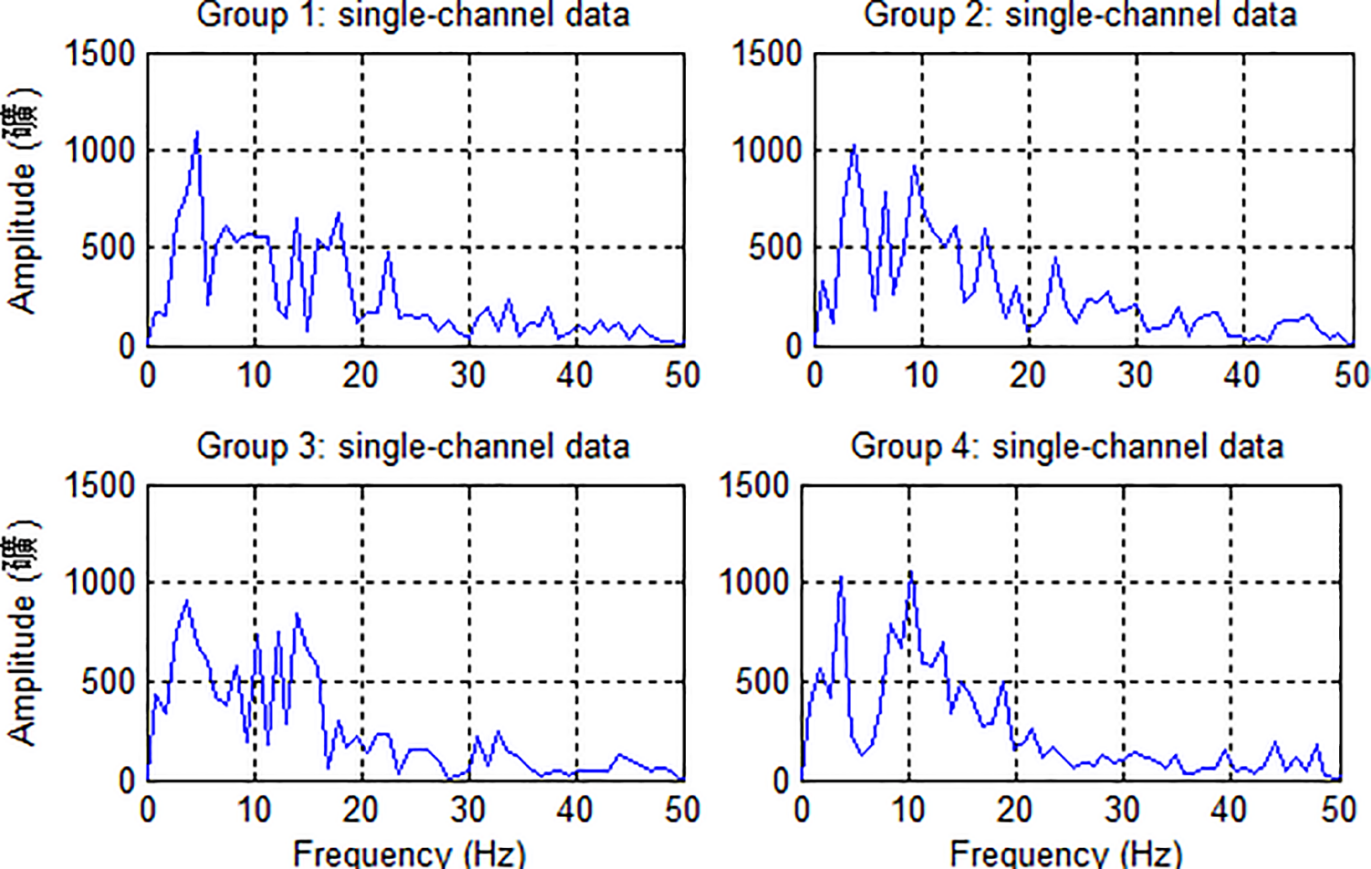
Amplitude spectra of the four single-channel (Oz) reference templates for a representative subject.

The analysis of frequency spectra is to find the frequency band on which the major power of c-VEP signals focused on. Frequency spectra of c-VEP signals were calculated by fast Fourier transform (FFT). Fig 6 shows the amplitude spectra of the four single-channel (Oz) reference templates (i.e. ERP signals) for a representative subject. It is clearly seen from the figure that although the amplitude spectra of the four ERP signals had different shapes, their major energy located basically in the frequency range of 2–40 Hz. Thus, temporally filtering the raw c-VEP signals in the frequency band can decrease the noise component and meanwhile improve their SNR to a certain degree.

To assess the SNR of c-VEP signals, four different methods for data preprocessing were explored, i.e. 1) M1: raw single-channel (Oz) signals without temporal filtering; 2) M2: single-channel (Oz) signals with temporal filtering; 3) M3: raw multi-channel signals without spatial filtering; 4) M4: multi-channel signals with both temporal and spatial filtering. M4 was the method used in the c-VEP BCI system. The SMI was evaluated using the training signal from each reference target and SNR was the average of the four SMIs derived from the four reference targets.

**Fig 7:**
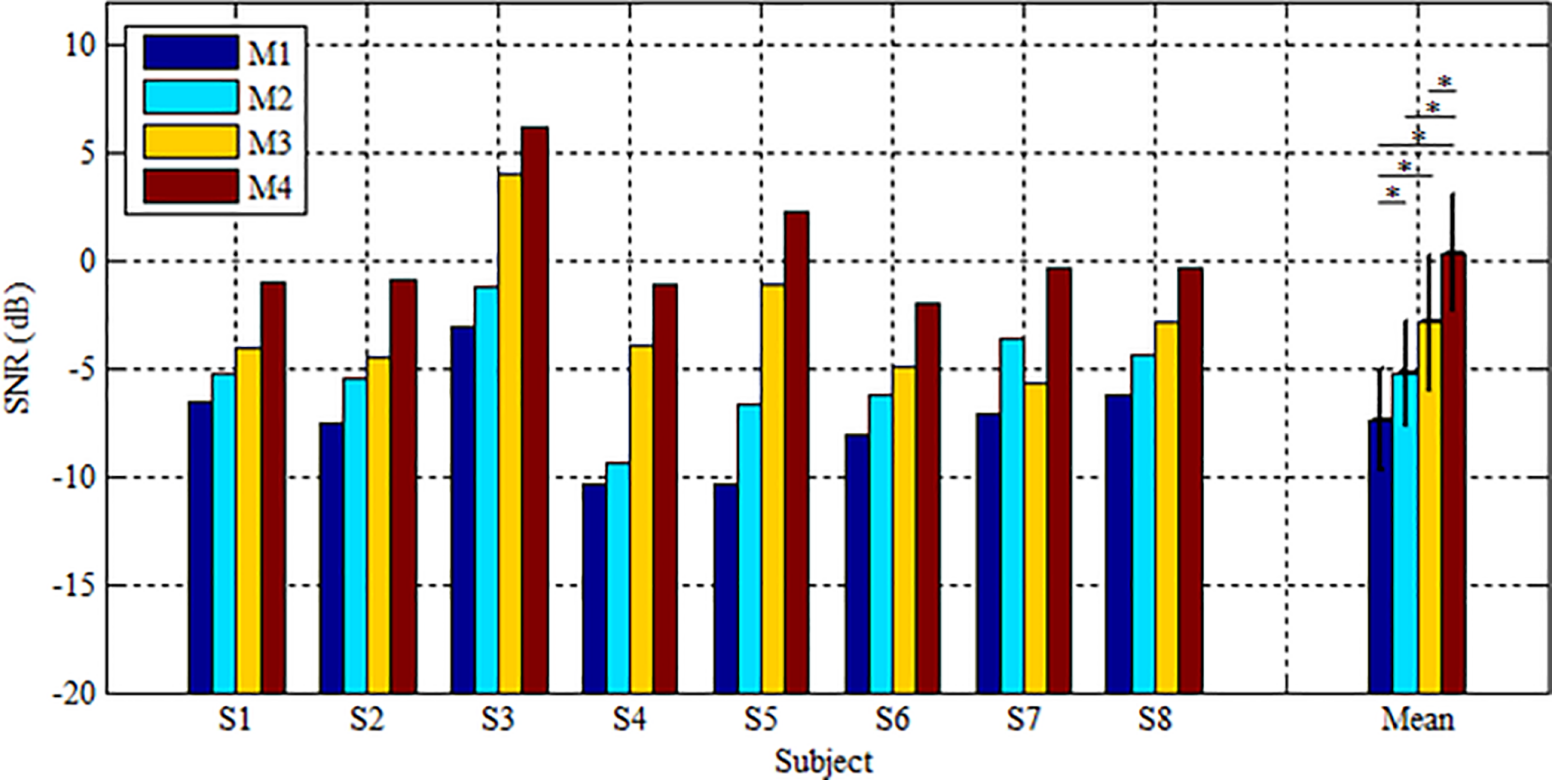
The averaged SNR of each subject across the c-VEP signals from the four reference targets and their mean for the four data processing methods M1~M4. The error bar denotes standard deviation. The asterisks on the far-right bar graph indicate the significant difference in SNR between any two of four methods obtained by Paired Wilcoxon signed-rank tests at 95% confidence level (*p<0.05).

Fig 7 shows the averaged SNR of each subject across the c-VEP signals from the four reference templates for the four data preprocessing methods. It exhibits that although the SNRs of the four methods varied from subject to subject, they behaved consistently for all subjects except for S7. On average, the poorest, moderate, good and the best SNRs were yielded by M1, M2, M3 and M4 respectively. Compared to the raw single-channel signal (M1), temporal filtering (M2) and spatial filtering (M3) increased solely the SNR of c-VEP signals, whereas their combination (M4) achieved the best SNR improvement. Paired Wilcoxon signed-rank tests at 95% confidence level showed that significant difference in SNR existed between any two methods except for M2 and M3 with p values all equaling 0.012.

### Correlation coefficient

**Fig 8:**
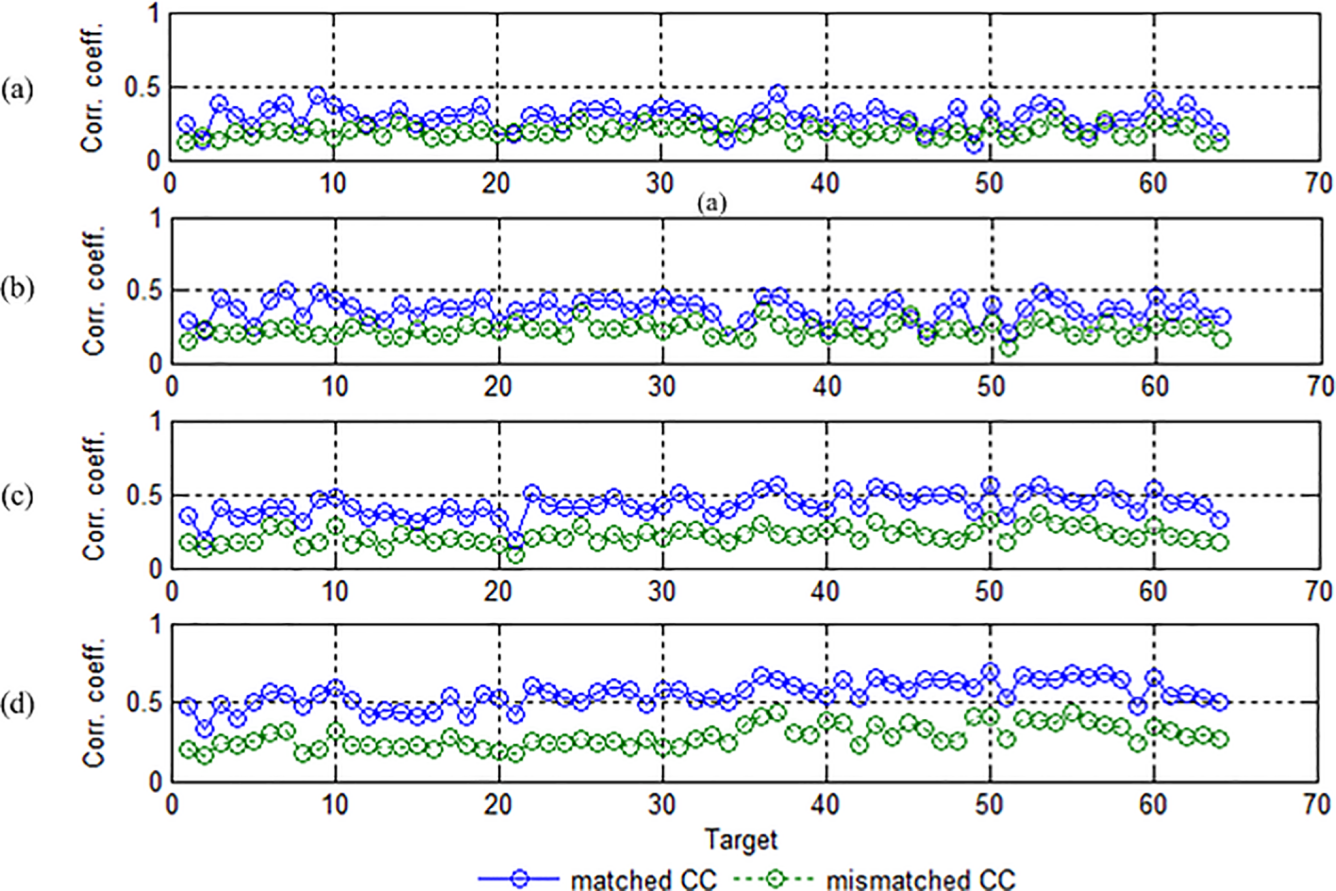
The averaged correlation coefficient across the five testing trials between each target and its template (*CC*_*T*_, blue cycles) and the averaged maximal correlation coefficient across the five testing trials between the same target and other templates (*CC*_*M*_, green cycles) for a representative subject. (a)~(d) correspond to the four data preprocessing methods M1~M4 respectively.

The correlation coefficients between each testing trial and all 64 templates were calculated for comparing differentiability of different targets. They included a matched correlation coefficient (*CC*_*T*_) yielded between the attended target and its template, and a maximal mismatched correlation coefficient (*CC*_*M*_) yielded between the same target and the other templates. Intuitively, a high detection accuracy required *CC*_*T*_ to be larger than *CC*_*M*_ for most stimulus target. Fig 8 shows *CC*_*T*_ (marked in blue) and *CC*_*M*_ (marked in green) derived from a representative subject for the four data preprocessing methods (M1~M4). Note that both *CC*_*T*_ and *CC*_*M*_ were averaged across the five testing trials of each target. Clearly, the distance between *CC*_*T*_ and *CC*_*M*_ denotes the separability of a testing target from the others. The farther the distance, the higher the detection accuracy of the target was.

In the first row, *CC*_*T*_ obtained from M1 was small for most targets, indicating that the detection accuracy was poor. In the second row, *CC*_*T*_ obtained from M2 was larger for most targets, but the distance between *CC*_*T*_ and *CC*_*M*_ was small, indicating that the temporal filtering in 2–40 Hz improved the detection accuracy to a certain extent. In the third row, both *CC*_*T*_ obtained from M3 and the distance between *CC*_*T*_ and *CC*_*M*_ were further increased for almost all targets, indicating that the spatial filtering with CCA significantly improved the detection accuracy. In the last row, both *CC*_*T*_ obtained from M4 and the distance between *CC*_*T*_ and *CC*_*M*_ were the largest for all targets, indicating that the best detection accuracy could be achieved by the combination of temporal and spatial filtering.

### Classification accuracy and ITR

The classification accuracy and ITR of single groups of targets and all the four groups of targets as a whole (Group w) are listed in Table 2. In terms of classification accuracies, the highest and the lowest values of the four target groups were 80% and 100% respectively. Although the eight subjects behaved differently, the averaged accuracies across subjects of the four target groups changed a little and all of them were higher than 90%. For most subjects, accuracy of a single target group was higher than that of Group w. On average, the accuracy yielded in Group w was obviously lower than that yielded in each single group, but was still as high as 88.36%. Paired Wilcoxon signed-rank tests showed that the accuracy for Group w was significantly lower than that for Group 2, 3 and 4 with *p* values equaling 0.021, 0.025 and 0.012 respectively, but there was no significant difference in accuracy between Group W and 1.

**Table 2:** Classification accuracy and ITR of single-group (Group 1, 2, 3 and 4) targets and the four groups of targets as a whole (Group w). The highest and lowest values are highlighted in bold face.

Subject	Classification accuracy (%)					ITR (bits/min)				
	Group 1	Group 2	Group 3	Group 4	Group W	Group 1	Group 2	Group 3	Group 4	Group W
S1	85	81.25	88.75	**80**	**75**	107.44	98.52	116.98	**95.66**	**141.55**
S2	91.25	96.25	98.75	98.750	92.50	123.76	138.80	147.67	147.67	197.98
S3	96.25	**100**	**100**	**100**	**98.44**	138.80	**153.26**	**153.26**	**153.26**	**221.86**
S4	83.75	95	95	90	81.88	104.40	134.80	134.80	120.32	162.22
S5	98.75	**100**	87.50	97.50	92.50	147.67	**153.26**	113.72	143.05	197.98
S6	83.75	97.50	100	96.25	88.44	104.40	143.05	153.26	138.80	183.61
S7	97.50	92.50	93.75	98.75	87.81	143.05	127.31	130.98	147.67	181.49
S8	95	88.75	97.50	93.75	90.31	134.80	116.98	143.05	130.98	190.11
Mean	**91.41**	**93.91**	**95.16**	**94.38**	**88.36**	**125.54**	**133.25**	**136.71**	**134.68**	**184.60**

With respect to ITR, the situation was just the opposite. Whether in terms of individual subjects or their average, the ITR yielded in Group w was significantly higher than that yielded in each single group, because the number of stimuli was largely increased from 16 to 64. Paired Wilcoxon signed-rank tests revealed that the ITR for Group w was significantly higher than that for group 1, 2, 3 and 4 with *p* values all equaling 0.012. Thereby, increasing the number of targets from 16 to 64 leads to small sacrifice in accuracy (91.41%, 93.91%, 95.16% and 94.38% versus 88.36%), but large gain in ITR (125.54 bits/min, 133.25 bits/min, 136.71 bits/min and 134.68 bits/min versus 184.6 bits/min). These results exhibit that the proposed grouping modulation paradigm is highly effective for improving the ITR of c-VEP BCIs by increasing the number of target groups.

## Discussion

This study aimed to massively increase the number of stimulus targets in c-VEP BCIs via grouping modulation of visual stimuli. Sixty four targets are divided into four target groups and each group of stimuli are modulated by a distinct pseudorandom binary code and its circularly shifting codes. In this way, only one target per group needs to be trained for creating templates of all targets in the group. Thereby, both the number of targets and the training time are increased with the number of groups. The difference is that the former is increased substantially because each group includes sixteen targets, whereas the latter is increased slightly because the training time per group is 1.066×100 = 106.6 s or 1.78 min for 100 training trials (i.e. 100 stimulation cycles). The total training time for all the four target groups is less than 8 minutes, which is rather small training time compared to that for motor imagery based BCIs [31]. By incorporating the two existing methods for target modulation mentioned in Introduction, the proposed c-VEP paradigm takes advantage of their strengths, i.e. the first method is used to reduce training time, whereas the second is used to raise the number of targets.

The number of available targets is a significant parameter in a BCI because a large number of targets are needed for some applications such as a word input system and for increasing ITR that is the most important metric for BCI performance. Thereby, an effort in BCI study is made to improve ITRs. In terms of f-VEP BCIs, many methods were developed for increasing the number of stimulus targets. Zhang et al. presented a novel protocol termed multiple frequency sequential coding (MFSC) [32], in which multiple frequencies are sequentially used in each cycle to code the target. To fulfill the sequential coding, each cycle is divided into several coding epochs and during each epoch, certain frequency is employed. The results suggested that the proposed protocol could potentially implement much more targets with the limited available frequencies. Jia et al. proposed a frequency and phase mixed coding approach to increase the number of targets and the resulting ITR [33]. An f-VEP BCI with fifteen targets was developed using the combination of three stimulation frequencies and five phases. Wang et al. presented an approximate method to render the visual stimulation with a high frequency resolution [34]. Any frequency lower than half of the screen refresh rate can be approximated by using variable frequencies in different stimulating periods. Using this approach, an f-VEP BCI with 16 targets was implemented and an average ITR of 75.4 bits/min was obtained. In this study, a grouping modulation paradigm is proposed to raise the number of targets to sixty four. This is truly meaningful for advancing the BCI technology from laboratory research to practical application.

ITR is determined by three parameters, i.e. classification accuracy, number of targets and detection time. A high ITR requires the first parameter to be high, the second to be large and the third to be short. However, these three parameters are interactive with each other instead of independent, making the design of BCI systems to be a complex task.

The high ITR yielded in this study is mainly attributed to the following factors: 1) a larger number of stimulus targets, 2) the four distinct modulation codes that have both good autocorrelation and good crosscorrelation property, 3) an efficient signal processing algorithm that incorporates temporal and spatial filtering, and 4) a short data segment used for target recognition. The proposed c-VEP BCI paradigm significantly facilitates the stimulus presentation of a large number of concurrent stimuli on a computer monitor. The visual stimuli yielded by the four modulation codes evoked near orthogonal c-VEP signals within a single group as well as between groups, making all targets easily distinguishable. The template matching based classification algorithm improved substantially the SNR of c-VEP signals and accurately recognized the vast majority of targets the subjects were attending to. The averaged classification accuracy across subjects is 88.36%, an accuracy rate much higher than the one (70%) for effective BCI communication [35]. Finally, the data segment used for classification of targets is only one stimulus cycle (approximately 1.066 s) that is relatively short compared to other BCI studies. All these factors together make the proposed c-VEP BCI to be a high-performance communication interface.

Although the proposed c-VEP BCI achieved a very high ITR, there is a room for improvement. In the present system, sixty four stimulus targets are presented on an area limited LCD monitor of size 24 inches (1920×1080 pixels) so that the size of each stimulus target is 140×100 pixels that is suboptimal for use in VEP BCIs. The previous studies [36–38] suggested that the optimal stimulus size is at least 170×170 pixels (visual angle of 3.8°) for a monitor of size 1920×1080 pixels. Therefore, increasing the size of each stimulus target will improve detection accuracy and the resulting ITR. Taking account of individual difference, selection of subject specific parameters such as channel location, filter band and the number of training trials can also be useful for improving individual performance.

The data length used for target recognition is one stimulus cycle, i.e. 1.066 s for the screen refresh rate of 60 Hz. The disadvantage of using the fixed stopping (FS) strategy is that it does not consider the individual discrepancies of subjects in BCI capacity. Even for the same subject, the BCI capacity can vary with time because the brain state is always changing. Thereby, it would be better to classify targets using different data length determined by the quality of EEG features and the classification confidence for each trial, i.e. adopting a dynamic stopping (DS) strategy. The DS strategy can not only improve the classification accuracy, but also shorten the overall time for target recognition. A recent study suggested that even for high-speed SSVEP BCIs, the DS strategy can further improve their performance [39]. Thus, incorporating the DS strategy into the 64-target c-VEP BCI would be a direction of future study.

## Conclusion

This study built a 64-target c-VEP BCI by grouping modulation of visual stimuli, in which all stimulus targets were divided into four groups and each group contained 16 targets that were modulated by a distinct pseudorandom binary code and its circularly shifting codes. Analyses of the experimental data from eight subjects indicated that at as average classification accuracy of 88.36%, an average ITR achieved by the multi-target BCI system was as high as 184.6 bits/min due to the large number of targets, an ITR much higher than that yielded by existing c-VEP BCIs, proving its great potential in some complex applications such as word inputting.
